# Alginate Hydrogel Assisted Controllable Interfacial Polymerization for High-Performance Nanofiltration Membranes

**DOI:** 10.3390/membranes11060435

**Published:** 2021-06-10

**Authors:** Zhao-Yu Ma, Yu-Ren Xue, Zhi-Kang Xu

**Affiliations:** MOE Key Laboratory of Macromolecular Synthesis and Functionalization, Key Laboratory of Adsorption and Separation Materials & Technologies of Zhejiang Province, Department of Polymer Science & Engineering, Zhejiang University, Hangzhou 310027, China; mazhaoyu@zju.edu.cn (Z.-Y.M.); yrxue4571@zju.edu.cn (Y.-R.X.)

**Keywords:** interfacial polymerization, nanofiltration, thin-film composite, alginate, hydrogel

## Abstract

The deepening crisis of freshwater resources has been driving the further development of new types of membrane-based desalination technologies represented by nanofiltration membranes. Solving the existing trade-off limitation on enhancing the water permeance and the rejection of salts is currently one of the most concerned research interests. Here, a facile and scalable approach is proposed to tune the interfacial polymerization by constructing a calcium alginate hydrogel layer on the porous substrates. The evenly coated thin hydrogel layer can not only store amine monomers like the aqueous phase but also suppress the diffusion of amine monomers inside, as well as provide a flat and stable interface to implement the interfacial polymerization. The resultant polyamide nanofilms have a relatively smooth morphology, negatively charged surface, and reduced thickness which facilitate a fast water permeation while maintaining rejection efficiency. As a result, the as-prepared composite membranes show improved water permeance (~30 Lm^−2^h^−1^bar^−1^) and comparable rejection of Na_2_SO_4_ (>97%) in practical applications. It is proved to be a feasible approach to manufacturing high-performance nanofiltration membranes with the assist of alginate hydrogel regulating interfacial polymerization.

## 1. Introduction

Water scarcity is one of the major challenges facing the world [1,2]. In addition to saving existing freshwater resources and recycling wastewater for reuse, the only way we can supplement freshwater is desalination beside the earth’s hydrological cycle [3]. Moreover, the quality of domestic water is gradually improving with the improvement of living standards [4]. Traditional microfiltration and ultrafiltration technologies can no longer meet the current growing demand for water purification [5]. Therefore, it is urgent to develop deeper-level membrane-based water purification technologies like nanofiltration [6,7].

At present, most of the membranes used for nanofiltration are thin-film composite (TFC) structures including non-woven fabric support layers, micro- or ultrafiltration porous layer, and the thin polyamide layer as the surface, which is the key to make the membrane selective [8]. The currently used polyamide layers for nanofiltration membranes are mostly semi-aromatic polyamides with about tens to hundreds nanometer thickness synthesized by piperazine (PIP) and trimesoyl chloride (TMC) at organic/aqueous interface through interfacial polymerization [9]. This method has been widely used since the ground-breaking discovery made by Cadotte and his coworkers in the 1970s [10]. Although membrane-based water purification technologies have an obvious advantage in practical applications, the performance of nanofiltration membranes has not yet reached people’s expectations, especially the trade-off effect between the water permeance and salt rejection, which restricts further breakthroughs in nanofiltration performance [11]. Therefore, the research interest has improved to find a way out of the dilemma, such as enhancing the water pathways in the polyamide layer by adding nanofillers [12,13,14,15], reducing the thickness of the polyamide layer through a free organic/aqueous interfacial polymerization [16,17] and especially controlling the interfacial polymerization to finely tune the physicochemical properties of polyamide layer [18,19,20].

People have struggled for high-performance nanofiltration membranes by all means. Livingston et al. proposed a breakthrough concept that introduces a sacrificial Cd(OH)_2_ nanostrands layer to store aqueous monomers [21]. These nanostrands can tune the release of amine monomers during interfacial polymerization to some extent. The thickness of the resultant polyamide layer was significantly reduced to sub-10 nm with fewer amine monomers diffusing to the reaction interface. Although the Cd(OH)_2_ nanostrands layer can be removed by hydrochloric acid after interfacial polymerization, it is still uneconomic and not environmentally friendly for water purification [22]. After that, inorganic nanomaterials [23,24,25], metal-organic framework nanoparticles [26,27], and cellulose nanocrystals [28] were demonstrated to construct an interlayer to further modify the porous substrate and suppress the diffusion of anime monomers. However, it is still a big challenge to evenly distribute or coat the nanowires or nanoparticles on a porous substrate with a large area or in a consecutive way. It has certain advantages that performing the interfacial polymerization at a free organic/aqueous interface. Firstly, much lower concentrations of amines and acyl chlorides can be used to obtain thinner polyamide nanofilms; the heat and the byproducts (hydrogen chloride, in most cases) of the reaction can be better released [16]. Nevertheless, the susceptible interface between two liquids makes it difficult to fabricate membranes uniformly with a large area.

**Figure 1:**
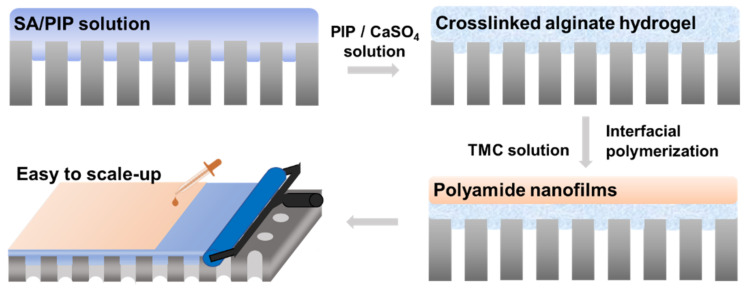
Schematic of an easy to scale-up approach to prepare nanofiltration membranes by building an alginate hydrogel reaction intermediated layer with a one-step crosslinking method.

Herein, we present a new strategy that using alginate hydrogel as the reaction intermediated layer to store the PIP monomers and to controllably release them during the interfacial polymerization (Figure 1). The high viscosity of the SA solution makes it easy to be evenly coated by a rubber roller at the porous substrate. And the SA chains can be cross-linked by Ca^2+^ by a one-step method (Figure S1) [29,30]. The established calcium alginate hydrogel layer can provide a stable platform to implement interfacial polymerization as well as store PIP monomers and release them controllably [18,31]. The non-fluidity property and restriction effect of the alginate hydrogel on amine monomers can significantly reduce the diffusion rate of PIP monomers resulting in thinner polyamide nanofilms than those prepared by conventional interfacial polymerization [32]. The as-prepared TFC membranes exhibit high water permeance (~30 Lm^−2^h^−1^bar^−1^) and comparable rejection of Na_2_SO_4_ (>97%) with the assisted alginate hydrogel reaction intermediate layer. Our work has improved the current problems of using the interlayer to prepare TFC membranes and paves the way to manufacture high-performance nanofiltration membranes by means of using natural polymers in a scalable and consecutive route.

## 2. Materials and Methods

### 2.1. Materials

Piperazine (PIP, 99%) and 1,3,5-benzenetricarbonyl trichloride (TMC, 98%) were purchased from Sigma-Aldrich, St. Louis, MO, USA. Sodium alginate (SA, AR) was obtained from Shanghai Makclin Biochemical Co., Ltd., Shanghai, China. Calcium sulfate anhydrous (CaSO_4_, CP), sodium sulfate (Na_2_SO_4_, 99%), sodium chloride (NaCl, 99.5%), magnesium sulfate (MgSO_4_, 98%), magnesium chloride (MgCl_2_, 98%), potassium chloride (KCl, 99.5%), sodium hydrate (NaOH, 96%), hydrochloric acid (HCl, 36–38%), sodium citrate dihydrate (99%), N,N-dimethylformamide (99.5%), dichloromethane (99.5%) and n-hexane (97%) were procured from Sinopharm Chemical Reagent Co. Ltd., Shanghai, China. Polyethersulfone microfiltration substrate (average pore size: 0.22 μm) was purchased from Haining Xindongfang Technology Co. Ltd., Haining, China. All the materials were used as received without further treatment. Ultrapure water (18.2 MΩ) used in all experiments was produced by an ELGA LabWater system (VWS Ltd., High Wycombe, UK).

### 2.2. Fabrication of Nanofiltration Membranes

The nanofiltration membranes were all prepared by interfacial polymerization using a homemade mold (15 × 15 cm^2^, 144 cm^2^ effective area) to clamp the substrate. The polyethersulfone porous substrate was soaked into ultrapure water for at least 72 h before use. The cleaned porous substrate was fixed on the mold, then 25 mL of SA and PIP mixed solution was poured evenly on the substrate to allowed the alginate hydrogel precursor to permeate into the porous substrate for 10 min. Then a rubber roller was used to remove the excess liquid on the surface of the substrate. Another 25 mL of CaSO_4_ and PIP mixed solution was added slowly on the substrate standing for 20 min. The concentration of PIP was kept the same between two mixed solutions to ensure the consistent PIP content in the resultant hydrogel layer. To reduce the cross-linking time, the concentration of CaSO_4_ was fixed at 2 g/L, which is slightly lower than its saturated solubility in water. Then the aqueous solution was collected for reusing and the substrate with calcium alginate hydrogel intermediated layer was allowed to air-dried to be with no obvious water droplets. The interfacial polymerization was implemented by adding 25 mL of TMC in n-hexane solution to react with PIP stored in an alginate hydrogel layer for 2 min. Then the as-prepared membrane was air-dried after removing the excess organic solution and was further cured under 60 °C for 15 min. The as-prepared membranes with different concentration of PIP (the concentration of SA was fixed at 15.5 g/L) or SA (The concentration of PIP was fixed at 1.5 g/L) for establishing hydrogel layer were denoted as PIP-0.5, PIP-1.0, PIP-1.5, PIP-2.0, PIP-3.0, and SA-3.8, SA-7.8, SA-11.6, SA-15.5 (SA–TFC), respectively. And a control group (TFC−Control) was prepared under the same conditions without a hydrogel layer. The concentration of TMC solutions were kept at a mass concentration of 1:1 with the concentration of PIP solutions in all experiments. All the membranes were thoroughly washed by ultrapure water to remove residuals and kept in a refrigerator at 4 °C before tests.

### 2.3. Membrane Characterization

The surface morphology of as-prepared membranes was measured under an accelerating voltage of 10 kV by field-emission scanning electron microscope (FESEM, Hitachi S4800, Tokyo, Japan). The samples were coated by a thin platinum layer by a magnetron target ion sputter coater (MSP 2S IXRF Systems Inc., Austin, TS, USA). Scanning probe microscopy (CSPM5500, Being Nano-Instruments, Guangzhou, China) was used to characterize the thickness and surface roughness of the membranes by working as atomic force microscopy (AFM) at tapping mode. The AFM samples were prepared by an improved method based on reported literature (Figure S2). In general, the SA–TFC membrane coupons were placed on the surface of cleaned silicon wafers. N,N-Dimethylformamide was used to dissolve the polyethersulfone porous substrate by fully soaking. Then the silicon wafers were washed by ultrapure water and immersed in the sodium citrate solution (100 g/L) to decrosslink the calcium alginate hydrogel to obtain the pure polyamide nanofilms.

The chemical compositions of the membranes were detected by FT-IR/ATR spectroscopy (Thermo Nicolet 6700, Thermo Fisher Scientific Co., Ltd., Shanghai, China) with an ATR accessory (ZnSe crystal, 45°) and X-ray photoelectron spectroscopy (XPS, Thermo Scientific K-Alpha, Thermo Fisher Scientific Co., Ltd., Shanghai, China) with Al-Kα excitation radiation (1486.6 eV). DropMeter A-200 contact angle testing system (MAIST Vision Inspection & Measurement Co. Ltd., Ningbo, China) was applied to measure the water contact angles and drop CDs of the membranes. The surface Zeta potential was characterized by a streaming potential analyzer (SurPASS, Anton Paar GmbH, Graz, Austria) using NaOH and HCl solutions to regulate the pH of electrolyte solution (KCl, 1 mmol/L).

### 2.4. Nanofiltration Performance Test

Nanofiltration performance was tested using a lab-scale cross-flow flat membrane filtration apparatus with a circular effective filtration area of 7.07 cm^2^. A membrane sample was subjected to a pre-compacted pressure of 6 bar for 30 min before the measurement of water permeance and salt rejection under an applied pressure of 4 bar. The involved salt solutions were all 2.0 g/L (2000 ppm) with an agitating speed of 300 rpm during the tests. All the solutions were maintained at 30 °C with a cross-flow rate of 25 L/h.

The water permeance (*P_w_*) of the membranes was calculated by:

(1) $$P_{w}=\frac{V}{AtP}$$

where *V* is the volume of permeated solution (L), *A* is the effective filtration area (m^2^), t is the permeation time (h), *P* is the applied pressure (bar) during the measurement.

The salt rejection was determined by

(2) $$R=\left(1-\frac{C_{p}}{C_{f}}\right)\times 100\%$$

where *C_p_* and *C_f_* are the concentrations of permeated and feed solutions, respectively. The concentrations of solutions were inferred by the conductivity of the solutions using a conductivity meter (METTLER TOLEDO, FE38, Shanghai, China).

The water/Na_2_SO_4_ permselectivity (A/B) was calculated by

(3) $$A/B=\frac{P_{w}}{P_{w}\left(1-R\right)P}$$

## 3. Results and Discussion

### 3.1. Physical Structures of the SA–TFC Membranes

**Figure 2:**
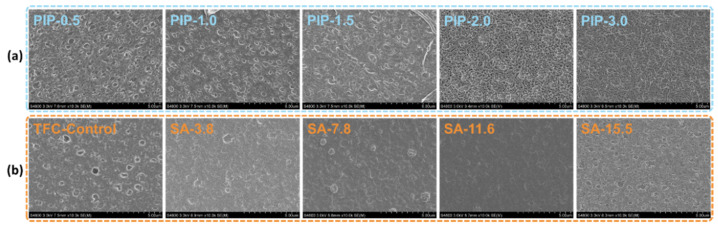
Surface topography of SA–TFC membranes synthesized at different concentrations of (**a**) PIP (SA is fixed at 15.5 g/L) and (**b**) SA (PIP is fixed at 1.5 g/L).

The surface morphology of resultant membranes was observed by FESEM, and the images were shown in Figure 2. The concentration of SA was fixed at 15.5 g/L at first to guarantee the stability of the hydrogel layer to perform interfacial polymerization. The surface was clean and flat until a higher concentration of PIP (>2 g/L) was used. It is noted that the resultant polyamide nanofilms are relatively thin so that the microporous contour of the polyethersulfone substrate can be observed when the concentration of PIP is lower than 1.5 g/L. It seems that the concentration of SA for constructing the hydrogel layer have little influence on the surface morphology of as-prepared SA–TFC membranes. But the difference between TFC−Control and SA–TFC is distinct with the smoother surface by introducing an alginate hydrogel layer. It is worth noting that the nodular structure may generate when the concentration of SA is low as the low-viscous SA aqueous layer is not stable at the crosslinking step leading to an uneven calcium alginate hydrogel layer. Also, the concentration of SA higher than 15.5 g/L is not used due to a liquid layer with visible wrinkles will generate after the roller coating step.

**Figure 3:**
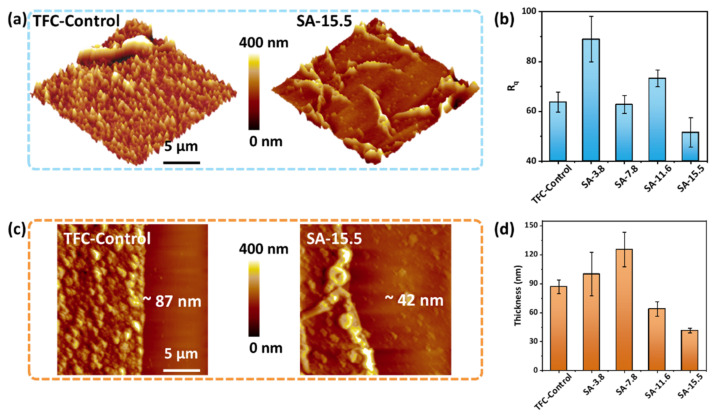
AFM results of polyamide nanofilms isolated from SA–TFC membranes. (**a**) Comparison of surface roughness of TCF-Control and SA-15.5; (**b**) The variations of the roughness of the polyamide nanofilms prepared at different concentration of SA; (**c**) AFM height images of TCF-Control and SA-15.5; (**d**) The changes of thickness of the polyamide nanofilms prepared at different concentration of SA.

The thickness and roughness of the polyamide nanofilms on the top of TFC−Control and SA–TFC membranes were detected by AFM at tapping mode with the same area of 20 × 20 μm. Figure 3a shows the variations of the surface morphology of TFC−Control and SA-15.5 membranes. The significantly reduced roughness is reflected in the transformation of the former serried nodules to sparse ribbons. Figure 3b indicates that there is no obvious change in the roughness of polyamide nanofilms prepared at different concentrations of SA. Despite the probable influence during the isolated nanofilms are transformed to a silicon wafer, the main cause of a rougher surface, especially at lower SA concentration, is that the roller-coated SA aqueous layer with lower viscosity is delicate to be washed out unevenly by the Ca^2+^ and PIP mixed solution in the next crosslinking step. Figure 3c shows the representative AFM images of the isolated polyamide nanofilms from TFC−Control and SA-15.5 membranes, which are loaded on the silicon wafer and cut by the scalpel. Although it may exist a small amount of residual hydrogel remaining on the bottom surface of the polyamide nanofilms, the overall thickness of the nanofilm decreases from 87 nm to 42 nm with the assist of alginate hydrogel during interfacial polymerization. There is a clear downward trend in the thickness of the polyamide nanofilms as the increasing concentration of SA in the alginate hydrogel layer results from a stronger restrictive effect on the diffusion process of PIP monomers (Figure 3d, Figures S3 and S4).

### 3.2. Chemical Composition of the SA–TFC Nanofiltration Membranes

**Figure 4:**
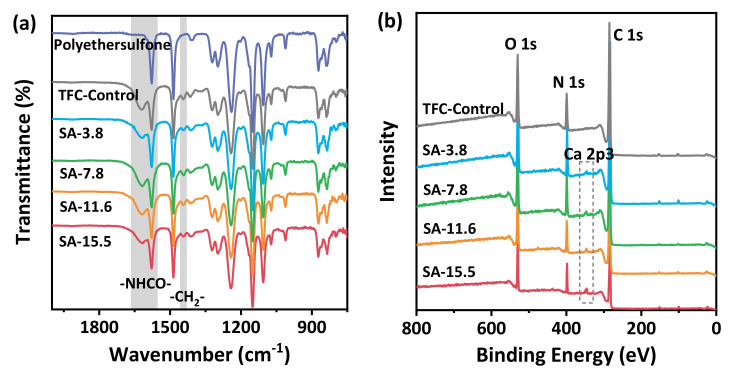
Chemical composition of the SA–TFC membranes: (**a**) ATR-FTIR spectra of the porous substrate, TFC−Control, and SA–TFC membranes; (**b**) XPS spectra of TFC−Control and SA–TFC membranes.

The chemical structure of resultant TFC membranes was analyzed by ATR-FTIR and XPS. Figure 4a shows the surface ATR-FTIR spectra of microporous substrates, TFC−Control, and SA–TFC membranes. The stretching vibration of C=O and the bending vibration of N-H are located at 1620 cm^−1^ (amide I peak) and 1580 cm^−1^ (amide II peak), respectively. The less intense peak at 1440 cm^−1^ is assigned to the methylenes (-CH_2_-) on the ring of PIP molecules. These characteristic peaks indicate that polyamide nanofilms can be successfully synthesized at the calcium alginate hydrogel surface and the absorption peak intensity of these polyamide nanofilms prepared with different concentrations does not change significantly under the sensitivity of infrared spectroscopy.

A further investigation of the elementary composition of the polyamide layer was conducted on XPS. Figure 4b shows the XPS full spectra of TFC−Control and SA–TFC membranes ranging from 0–800 eV. It is noted that a small amount of calcium can be detected in the SA–TFC membranes. According to the peak fitting results (Figures S4–S8) and integrated comparison of element contents (Table 1), it can be inferred that small amounts of elements in the alginate hydrogel layer under the surface can be detected when the polyamide nanofilm is very thin. And as the concentration of SA increases, the subsequent calcium ions used as cross-linking points are more fixed on the surface of the gel layer. Furthermore, with the increase of the SA concentration, the O/N ratio of the polyamide film has a downward trend, and the theoretical crosslinking degree has also increased, indicating that the SA gel layer has a regulatory effect on the reaction process by controlling the diffusion process of PIP and affects the general structure of the resulting polyamide nanofilms.

### 3.3. Surface Properties of the SA–TFC Nanofiltration Membranes

**Figure 5:**
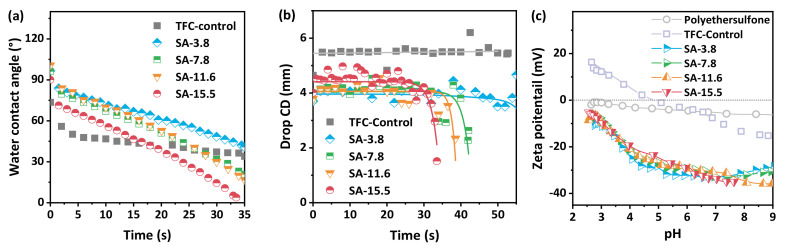
Surface properties of the SA–TFC membranes: (**a**) Dynamic water contact angle of TFC−Control and SA–TFC membranes; (**b**) Dynamic drop CD of TFC−Control and SA–TFC membranes; (**c**) Zeta potential of the porous substrate, TFC−Control, and SA–TFC membranes.

The surface properties of the polyamide nanofiltration membranes determine the separation properties to a certain extent. The dynamic water contact angle (Figure 5a) and drop CD (Figure 5b) were recorded to analyze the hydrophilicity of TFC−Control and SA–TFC membranes. It can be seen that although the initial water contact angles of SA–TFC groups are slightly higher than that of TFC−Control, the water droplets on it can spread fast and penetrate to the backside of the membranes in a very short time. Moreover, the higher the concentration of SA is used for the alginate hydrogel layer, the faster the penetration process becomes. The strong water affinity of alginate itself is conducive to the rapid water penetration process. Meanwhile, this is also ascribed to the overall reduction in the thickness of polyamide nanofilm synthesized on the alginate hydrogel layer.

Then the surface charge properties of the membranes were detected by a potentiometric analyzer at various pH values. The comparison of the results (Figure 5c) shows that the surface potential of the outermost polyamide nanofilms will decrease from about −8 mV to −33 mV even under the condition of an alginate hydrogel layer with a lower SA concentration. However, it barely has an effect on the negative charge of the membranes with an increasing concentration of SA to construct the hydrogel intermediated layer. Combined with the dynamic contact angle results, the initial contact angles among the four groups of SA–TFC membranes are almost the same but there are significant differences in the subsequent infiltration process, it can basically be inferred that changing the concentration of SA in the hydrogel layer will hardly affect the outermost part of the polyamide nanofilm. Nevertheless, it can actually regulate the subsequently generated structure of polyamide nanofilms by reducing the density and overall thickness to realize a faster water penetration process.

### 3.4. Nanofiltration Properties of the SA–TFC Membranes

**Figure 6:**
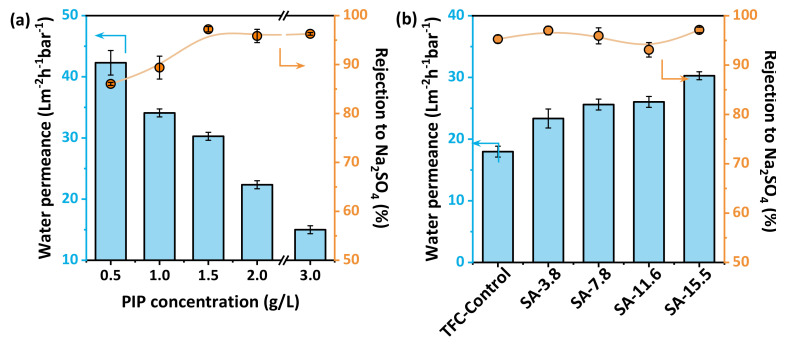

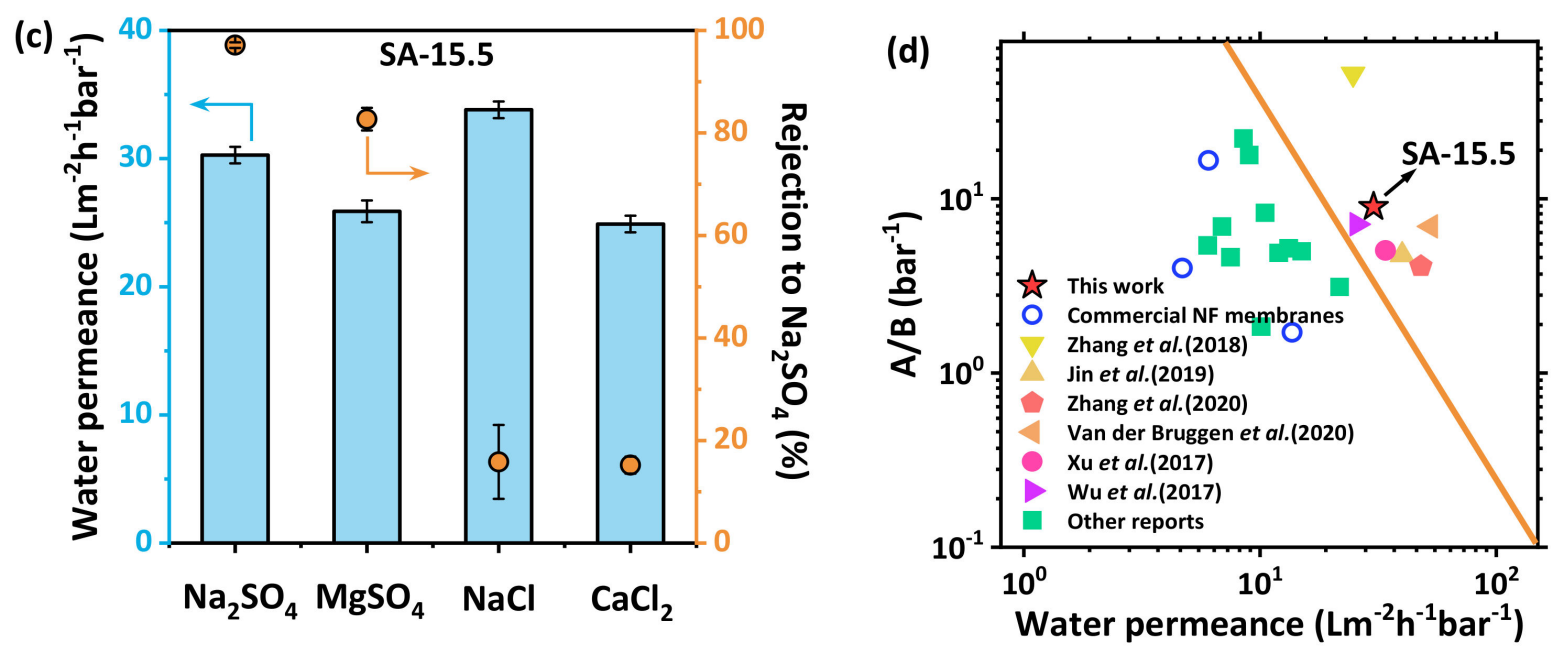
Nanofiltration properties of the SA–TFC membranes: (**a**) The Na_2_SO_4_ separation properties of SA–TFC membranes synthesized at different concentration of PIP (SA is fixed at 15.5 g/L); (**b**) Nanofiltration performance of SA–TFC membranes; (**c**) Separation performance of SA-15.5 membranes for different salt solutions; (**d**) Summary of water permeance and water/Na_2_SO_4_ permselectivity (A/B) of SA-15.5 and other high-performance nanofiltration membranes prepared by interfacial polymerization. Our membranes were tested under 4 bar operating pressure and cross-flow velocity of 25 L h^−1^ at 30 °C with 2 g L^−1^ salts as feed.

The nanofiltration performance was assessed in a cross-flow flat membrane module at 4 bar. The heat treatment time is optimized to 15 min considering the influence of the hydrogel layer on the drying process after the interfacial polymerization. Figure 6a shows that the water permeance of the resultant TFC membranes decreases from 42.3 Lm^−2^h^−1^bar^−1^ to 15.0 Lm^−2^h^−1^bar^−1^ with the increase of the concentration of PIP when the concentration of SA is fixed at 15.5 g/L, while the rejection of Na_2_SO_4_ increases to above 95% with a higher concentration of PIP than 1.5 g/L is used. Therefore, the optimized concentration of PIP is fixed to 1.5 g/L, and the initial concentration of SA for establishing the hydrogel layer is adjusted to control the nanofiltration properties of polyamide nanofilms. Figure 6b reveals that the rejection of Na_2_SO_4_ maintains around 97%, but the water flux is greatly increased (about 70%) comparing with the TFC−Control group as the concentration of SA in the hydrogel layer increases, that is the reduced overall thickness of polyamide layer facilitates the improved water permeance. performance of nanofiltration membranes is determined by steric and Donnan effects. The separation performance for different types of mono/divalent salts is basically in line with the characteristics of nanofiltration (Figure 6c), which exhibits a rejection order: Na_2_SO_4_ (97.2%) > MgSO_4_ (82.7%) > CaCl_2_ (15.8%) ≈ NaCl (15.2%). It also has certain advantages in breaking the trade-off effect compared with other high-performance nanofiltration membranes recently reported in the literature [14,19,24,27,28,32,33,34,35,36,37,38,39,40,41,42,43,44,45,46,47,48]. The collected water permeance and water/Na_2_SO_4_ permselectivity A/B are shown in Figure 6d and Table 2.

## 4. Conclusions

In conclusion, we have demonstrated a feasible way to fabricate high-performance nanofiltration membranes by using SA to establish an alginate hydrogel layer on the porous substrates. The Ca^2+^ crosslinked hydrogel layer can provide a steady platform to implement interfacial polymerization and work as an aqueous phase to store PIP monomers and release them during the reaction with confined diffusion. A smoother surface (R_q_ = 51 nm) than traditional TFC control group (R_q_ = 64 nm) is achieved. The thickness of resultant polyamide nanofilms are reduced from 87 nm to 42 nm with a controlled diffusion process of PIP. Therefore, an increase of 68% in water permeance compared to the TFC−Control group (30.27 Lm^−2^h^−1^bar^−1^) and comparable rejection (97.2% to Na_2_SO_4_) are achieved based on a thinner polyamide layer combining with a more negatively charged surface. To this point, we present an achievable method to fabricate advanced nanofiltration membranes with the assist of alginate hydrogels. It shows the potential for large-scale preparation by using a natural product just with easy steps in traditional TFC membrane fields.

## Figures and Tables

**Table 1:** Surface composition of polyamide layer on TFC−Control and SA–TFC membranes.

Sample	Element Content (Atomic, %)				O/N	Theoretical Degree of Cross-Linking (%) ^1^
	C	N	O	Ca		
TFC−Control	71.80	10.76	17.44	/	1.62	29.01
SA-3.8	73.75	10.47	15.86	0.33	1.51	39.06
SA-7.8	74.48	10.26	14.85	0.41	1.45	44.84
SA-11.6	73.41	10.54	15.49	0.56	1.47	42.92
SA-15.5	71.48	11.52	16.04	0.96	1.39	53.19

^1^ Calculating method based on the reference [21].

**Table 2:** The summary of detailed nanofiltration performance of SA-15.5 and other high-performance nanofiltration membranes in Figure 6d.

Ref.	Water Permeance (Lm^−2^h^−1^bar^−1^)	Rejection to Na_2_SO_4_ (%)	A/B	Operating Pressure (bar)	Salt Concentration (g/L)
SA-15.5	30.27	97.2	8.9	4	2
NF90 [33]	4.69	95	4	5	3.55
NF270 [34]	13.68	96.1	1.71	15	0.99
Desal-5 DK [35]	6.05	99.6	16.6	15	0.99
[19]	24.79	99.6	52.07	4.8	2
[24]	40	96.5	4.76	6	1
[27]	48	93.9	4.1	4	1
[32]	52.8	96.4	6.94	4	1
[36]	15	98	5	6	1.42
[28]	34	96.7	5.05	6	1
[37]	25.7	96	7.14	3.5	1
[38]	21.7	84	3.125	2	1
[39]	9	98.6	17.85	4	2
[40]	12	96.6	4.9	6	2
[41]	13.2	96.8	5.21	6	1
[42]	7.5	96.4	4.63	6	1
[43]	6.9	97.6	6.94	6	2
[44]	10.5	98	8.33	6	2
[45]	10.1	91	1.85	6	1
[46]	8.5	99.1	22.22	5	1
[47]	20.4	95.6	7.58	3	1
[48]	6	96.3	5.41	5	1

## Data Availability

We confirm all data and images are available from the corresponding author upon request.

## Supplementary Materials

The following are available online at https://www.mdpi.com/article/10.3390/membranes11060435/s1, Figure S1: Molecular formula of SA and a schematic diagram of the fast sol-gel transformation of SA induced by CaSO_4_, Figure S2: Schematic illustration of the isolation process of polyamide nanofilms from an SA–TFC membrane, Figure S3: AFM images and related height profiles of the polyamide nanofilms isolated from TFC−Control and SA–TFC membranes, Figure S4: SEM cross-section images of TFC−Control and SA–TFC membranes, Figures S5–S9: XPS full spectrum and the C, N, O, Ca peak fitting spectra of TFC membranes, Figure S10: The influence of thermal treatment time on nanofiltration performance of SA-15.5.
