# Supplementary material for: Alginate Hydrogel Assisted Controllable Interfacial Polymerization for High-Performance Nanofiltration Membranes
Source: Membranes (Basel). 2021 Jun 10;11(6):435. doi: 10.3390/membranes11060435 (PMC8228237; doi:10.3390/membranes11060435)
Supplement: Supplementary file 1 [file membranes-11-00435-s001.zip › membranes-1247641-supplementary.pdf]

Supporting Information for

Alginate Hydrogel Assisted Controllable Interfacial Polymerization for High-Performance Nanofiltration Membranes

Zhao-Yu Ma, Yu-Ren Xue and Zhi-Kang Xu *

MOE Key Laboratory of Macromolecular Synthesis and Functionalization, Key Laboratory of Adsorption and Separation Materials & Technologies of Zhejiang Province, Department of Polymer Science & Engineering, Zhejiang University, Hangzhou 310027, China; mazhaoyu@zju.edu.cn (Z.-Y.M.); yrxue4571@zju.edu.cn (Y.-R.X.)

* Correspondence: zkxu@zju.edu.cn; Tel.: +86-571-87952605

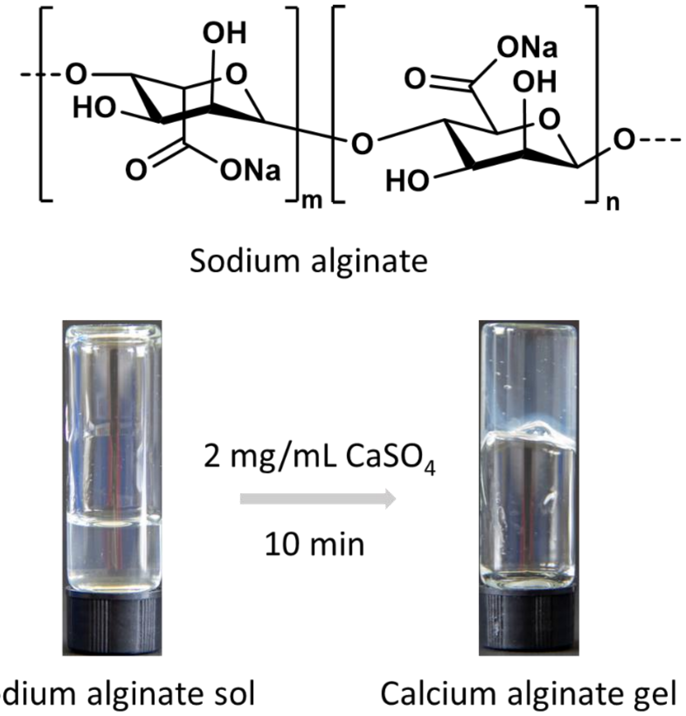

Figure S1. Molecular formula of SA and a schematic diagram of the fast sol-gel transformation of SA induced by $CaSO_4$.

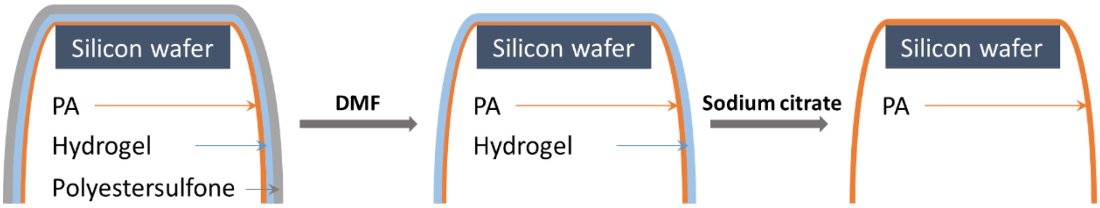

Figure S2. Schematic illustration of the isolation process of polyamide nanofilms from a SA-TFC membrane.

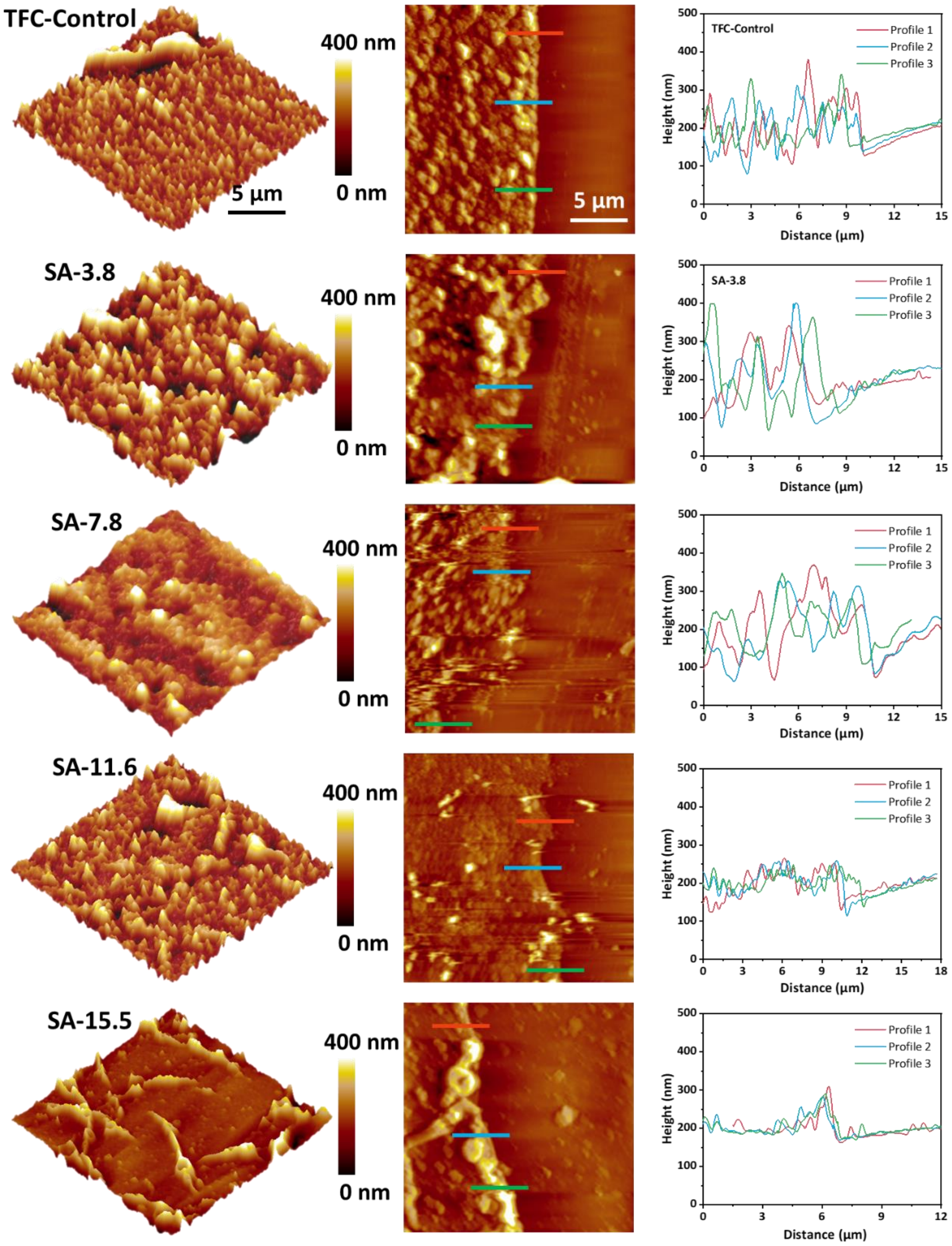

Figure S3. AFM images and related height profiles of the polyamide nanofilms isolated from TFC-Control and SA-TFC membranes.

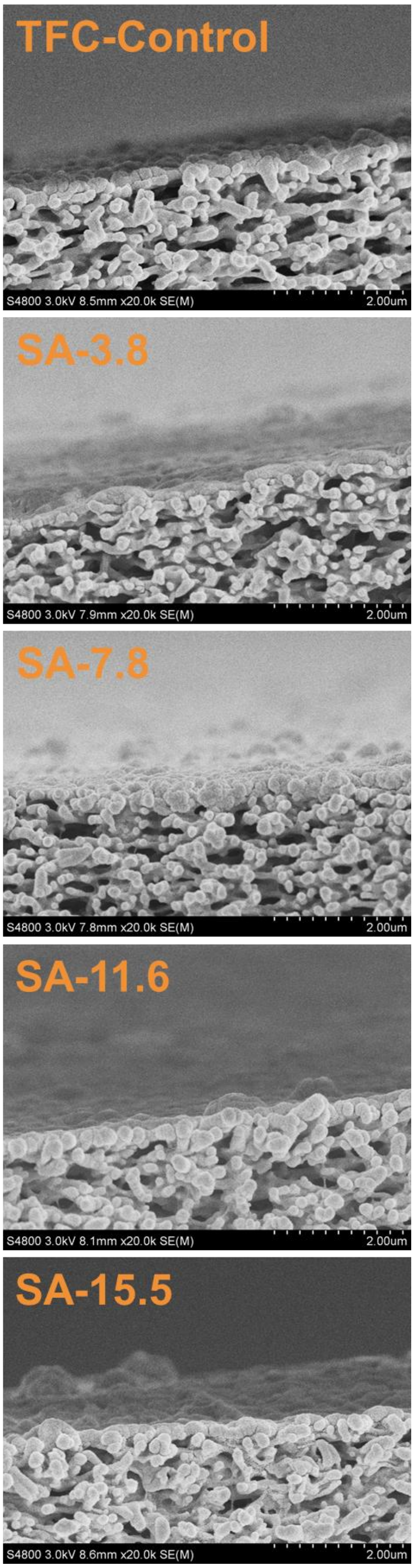

Figure. S4 SEM cross-section images of TFC-Control and SA-TFC membranes.

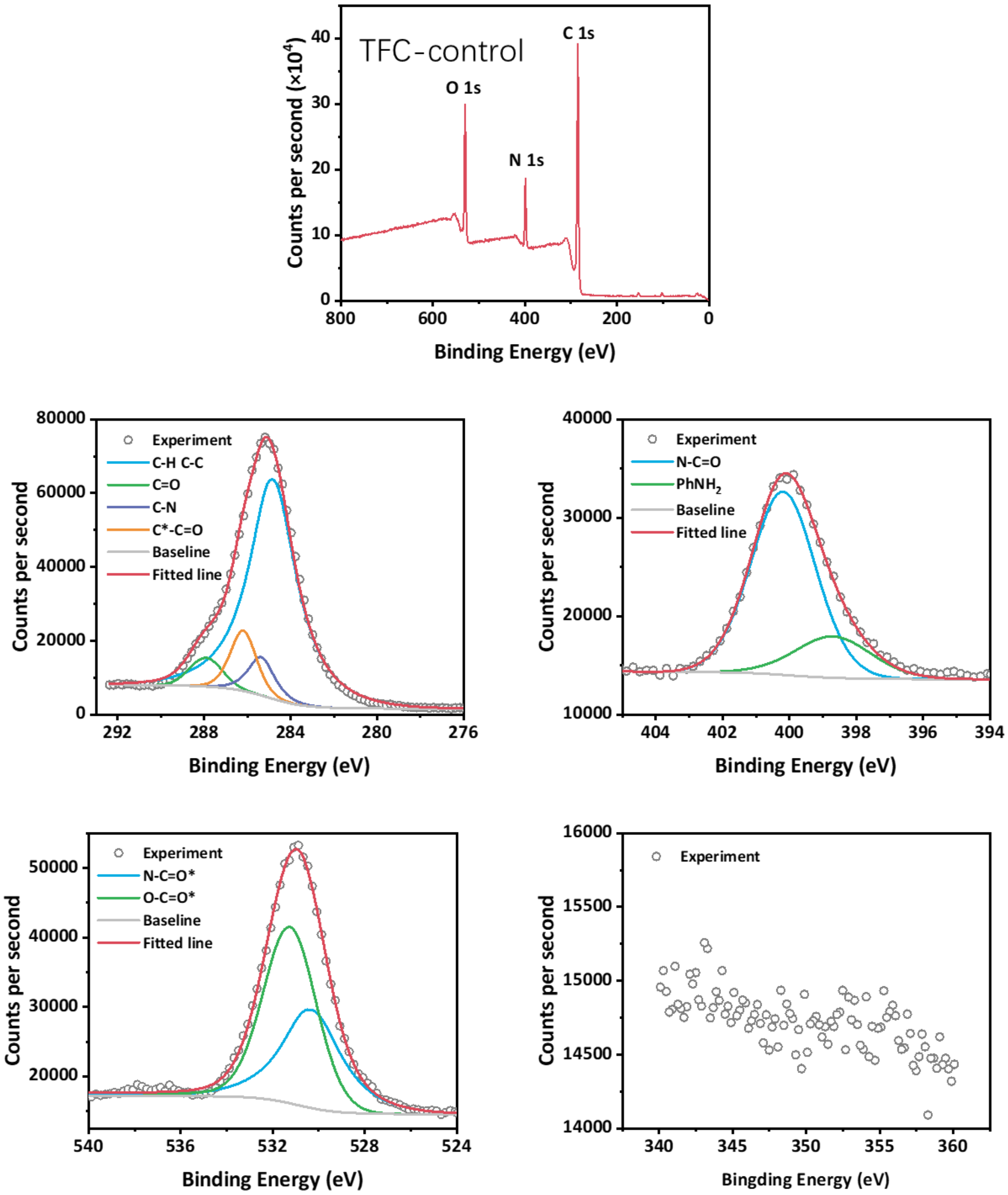

Figure S5. XPS full spectrum and the C, N, O, Ca peak fitting spectra of TFC-Control membrane.

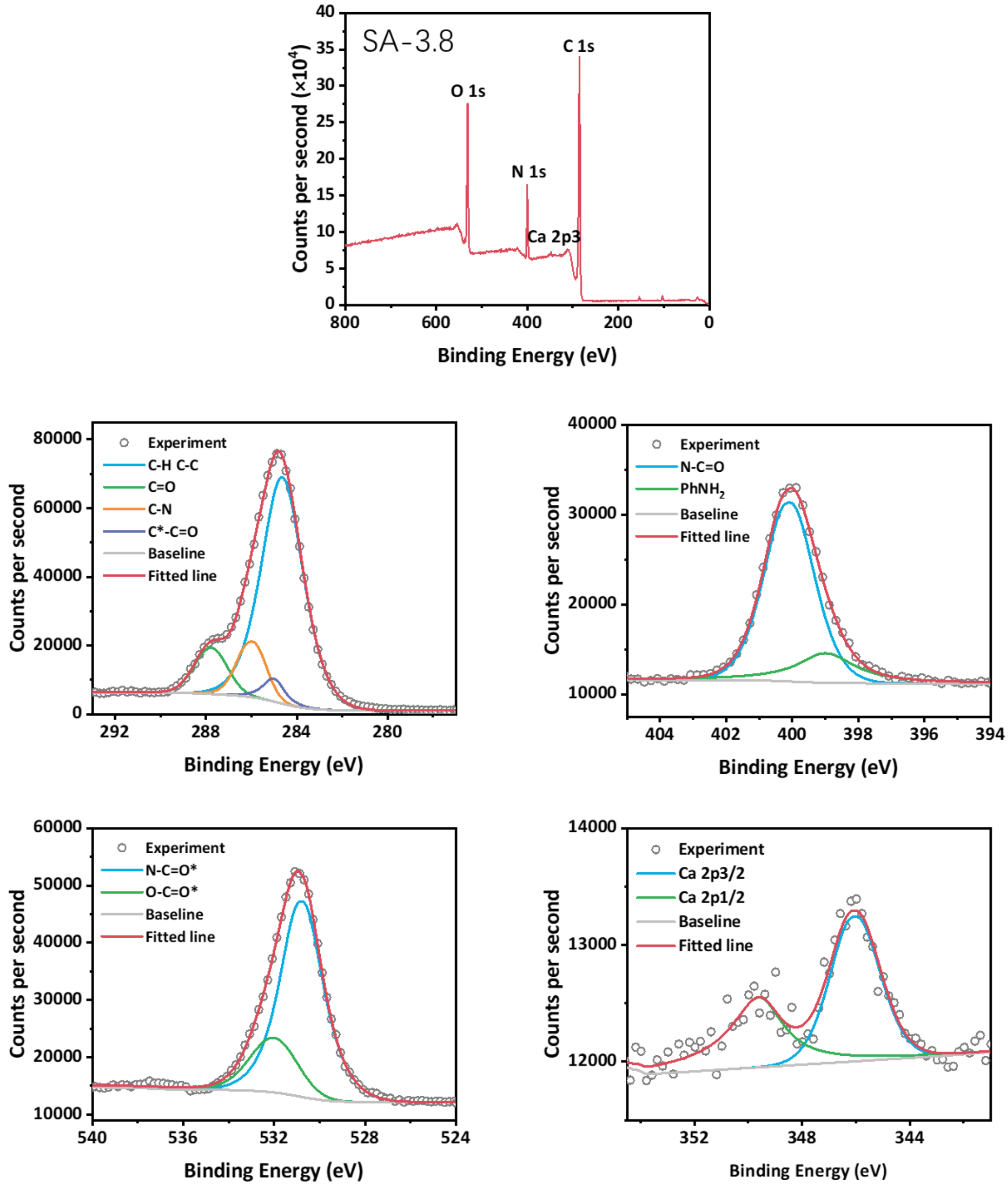

Figure S6. XPS full spectrum and the C, N, O, Ca peak fitting spectra of SA-3.8 membrane.

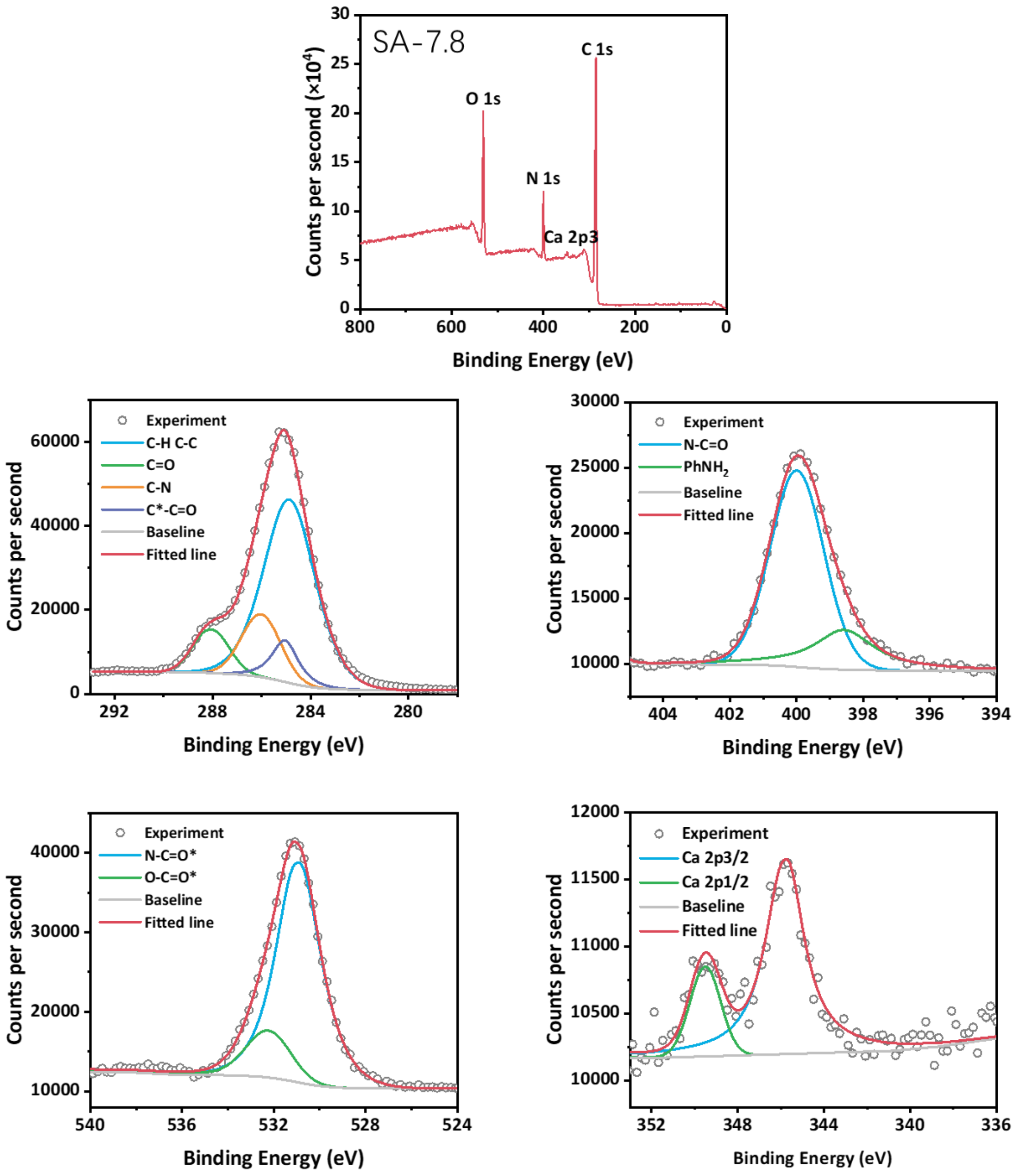

Figure S7. XPS full spectrum and the C, N, O, Ca peak fitting spectra of SA-7.8 membrane.

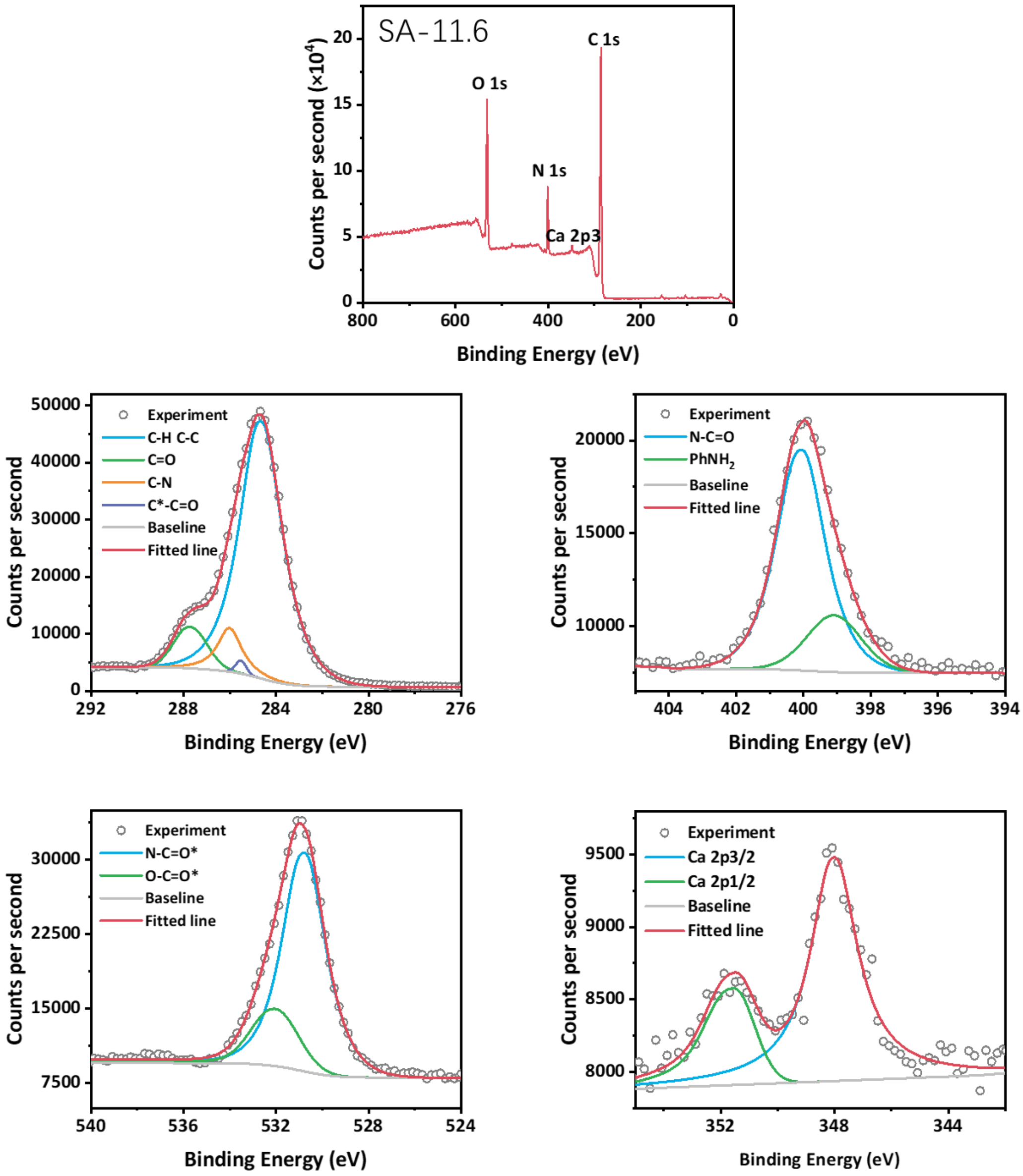

Figure S8. XPS full spectrum and the C, N, O, Ca peak fitting spectra of SA-11.6 membrane.

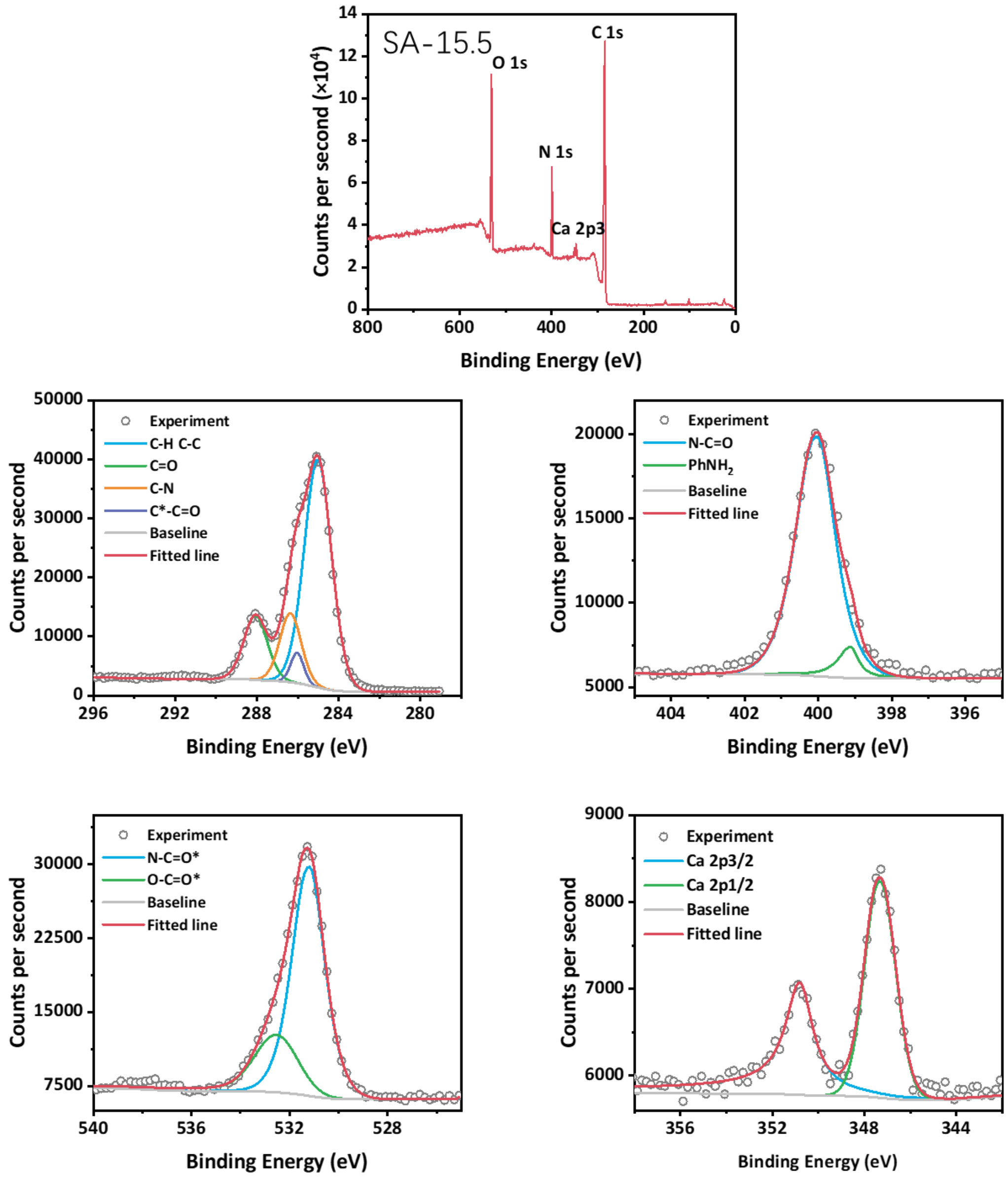

Figure S9. XPS full spectrum and the C, N, O, Ca peak fitting spectra of SA-15.5 membrane.

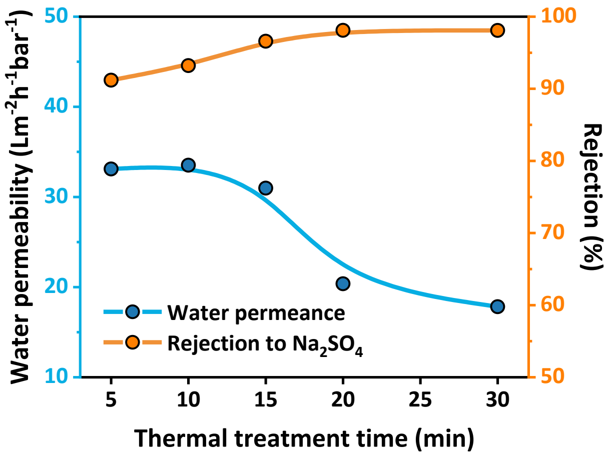

Figure S10. The influence of thermal treatment time on nanofiltration performance of SA-15.5
